# Age as a key risk factor for deep vein thrombosis in patients with lower limb cellulitis

**DOI:** 10.3389/fmed.2025.1567280

**Published:** 2025-06-20

**Authors:** Romane Teshima, Natsuko Saito-Sasaki, Yu Sawada

**Affiliations:** Department of Dermatology, University of Occupational and Environmental Health, Kitakyushu, Japan

**Keywords:** cellulitis, deep vein thrombosis, statistical analysis, risk factor, age

## Abstract

**Background:**

Cellulitis of the lower extremities is a common bacterial skin infection that can lead to various complications, including deep vein thrombosis (DVT). Although previous studies have suggested an increased risk of thrombosis due to the pro-inflammatory state induced by cellulitis, the specific clinical characteristics and risk factors associated with DVT in cellulitis patients remain poorly understood. This study aims to identify the clinical characteristics and risk factors for DVT in patients with lower limb cellulitis.

**Methods:**

We conducted a 10-year retrospective study of patients treated for lower limb cellulitis at our institution between January 2013 and December 2023. Patients who underwent ultrasound examination to assess for DVT were included in the analysis. Clinical data were collected, including age, D-dimer levels, C-reactive protein (CRP) levels, body mass index (BMI), duration of antibiotic treatment, and relevant medical history (e.g., diabetes, malignancy, the use of corticosteroids, and immunosuppressive agents). Statistical analyses were performed to identify risk factors for thrombosis.

**Results:**

Among the patients included in the study, those who developed DVT were significantly older, with the majority being 70 years or older. No significant associations were found between thrombosis and other clinical parameters, including elevated CRP levels, D-dimer levels, BMI, or the duration of antibiotic treatment. Additionally, neither diabetes nor the use of corticosteroids or immunosuppressants showed a significant correlation with DVT development. Importantly, thrombosis occurred in both the affected and non-affected limbs, with no significant difference between them.

**Conclusion:**

Age, particularly being 70 years or older, was identified as an independent risk factor for the development of DVT in patients with lower limb cellulitis. These findings suggest that advanced age plays a critical role in DVT development in cellulitis patients, and further investigation is needed to explore the underlying mechanisms.

## Introduction

Cellulitis is a common bacterial skin infection, particularly affecting the lower extremities (1). While the primary treatment focuses on addressing the underlying infection with antibiotics, there has been increasing attention to the potential complications that can arise in cellulitis patients, including the development of deep vein thrombosis (DVT) (2). Prior studies suggest that the pro-inflammatory state induced by cellulitis may elevate thrombotic risk (3, 4). However, the specific mechanisms and definitive risk factors remain unclear and subject to debate.

Deep vein thrombosis is a condition in which blood clots form in the deep veins and can lead to serious complications, including pulmonary embolism (5). Various clinical factors are known to predispose individuals to DVT, such as obesity, advanced age, malignancy, immobility, and elevated inflammatory markers (6–9). Given that cellulitis itself often leads to limb immobilization and an inflammatory response, it is plausible that these factors may act synergistically to increase thrombosis risk.

However, the relationship between cellulitis and DVT is not well established, and few studies have comprehensively examined the clinical characteristics that may contribute to thrombosis in this patient population (3, 4). Notably, the majority of prior studies have focused on Western populations, leaving a gap in understanding the risks specific to Asian populations, where thrombotic events are reportedly less frequent. This gap highlights the importance of investigating whether demographic differences influence thrombosis risk in cellulitis.

Additionally, while factors such as elevated C-reactive protein (CRP) levels, body mass index (BMI), and the use of corticosteroids are commonly associated with increased thrombosis risk (10–12), their specific roles in cellulitis-related thrombosis have not been clearly defined. Moreover, the influence of age and other demographic factors on thrombosis in cellulitis patients remains underexplored.

To address these gaps, this study aimed to investigate the clinical characteristics associated with the development of DVT in cellulitis patients. We hypothesized that certain clinical factors, such as elevated inflammatory markers would predispose patients to thrombosis, particularly in the affected limb. Furthermore, we focused on the Japanese population, where research on this topic has been limited, to explore the influence of demographic factors on thrombotic risk.

## Materials and methods

### Study design and population

This retrospective study was conducted on patients treated for lower limb cellulitis at the University of Occupational and Environmental Health between January 2013 and December 2023. The study population included individuals diagnosed with cellulitis and treated with antibiotics during this period. Among these, only patients who underwent ultrasound examination for DVT assessment, prompted by elevated D-dimer levels exceeding the normal range (D-dimer ≥ 0.5 μg/m), were included in the final analysis. In our cohort, all patients underwent D-dimer testing prior to venous ultrasonography, regardless of the level of clinical suspicion. There were no cases in which ultrasonography was performed without prior D-dimer measurement.

### Data collection

Clinical data were retrospectively extracted from medical records, including both numerical and categorical variables. The numerical data included age, D-dimer levels (ng/ml), CRP levels (mg/L), and BMI (kg/m^2^). Categorical variables included sex, medical history such as the presence of diabetes mellitus or malignancy, and the use of corticosteroids.

### Location of thrombosis

The anatomical location of thrombosis was determined based on ultrasound findings and categorized into the affected limb, corresponding to the limb with cellulitis, and the contralateral limb. This differentiation allowed for a detailed evaluation of thrombosis localization and its relationship to cellulitis.

### Outcome measures

The primary outcome measure of this study was the development of DVT in patients with lower limb cellulitis, as confirmed by ultrasound examination. Thrombosis was assessed in both the affected and contralateral limbs. Clinical characteristics, including age, D-dimer levels, CRP levels, duration of antibiotic treatment, BMI, and medical history, were compared between patients with and without DVT.

### Statistical analysis

Descriptive statistics were used to summarize the baseline characteristics of the study population. Continuous variables, such as age, D-dimer levels, CRP levels, BMI, and duration of antibiotic treatment, were reported as means with standard deviations or medians with interquartile ranges, depending on data distribution. Categorical variables, including sex, the presence of diabetes mellitus or malignancy, and the use of corticosteroids, were summarized as frequencies. For the purpose of further stratification, patients were categorized based on key clinical thresholds: CRP ≥ 3 mg/L, D-dimer ≥ 2 μg/ml, and BMI ≥ 25 kg/m^2^. Notably, the thresholds used for CRP (≥3 mg/L) and D-dimer (≥2 μg/ml) were selected based on previous studies and clinical relevance. A CRP level of 3 mg/L or higher has been used to define high inflammation states (13). For D-dimer, a threshold of ≥2 μg/ml was used to further stratify patients at higher thrombotic risk, as supported by prior studies showing increased DVT incidence at such levels (14).

Comparisons between patients who developed DVT and those who did not were performed using t-tests for continuous variables and Fisher’s exact test or Chi-square test for categorical variables. Univariate and multivariate logistic regression analyses were performed using the same variables listed in Table 1, which were selected based on their clinical relevance and previously reported associations with thrombotic risk: age over 70, sex, BMI, CRP, D-dimer, diabetes mellitus, cancer, and corticosteroid use.

**TABLE 1:** Clinical characteristics of the presence of DVT.

Variables	DVT (+)	DVT (−)	*P*-value
Number of cases	7	29	
Age (Range)	78.7 (61–89)	66.3 (37–84)	0.023
Sex			0.674
Male	5	16	
Female	2	13	
CRP (mg/dl) (range)	10.5 (0.14–21.8)	13.1 (0.11–35)	0.542
D-dimer (μg/ml) (range)	6.27 (1.6–15.1)	5.65 (0.8–37.7)	0.837
BMI average (kg/m^2^) (range)	25.9 (20.6–30.7)	30.8 (19.4–53.6)	0.227
Cancer	2	3	0.224
Corticosteroid use	2	8	>0.999
Diabetes	3	9	0.664

All statistical analyses, including descriptive statistics, group comparisons, and regression analyses, were performed using IBM SPSS Statistics version 27 (IBM, Chicago, IL, USA) and GraphPad Prism version 9.5.0 (GraphPad Software, San Diego, CA, USA). A *P*-value of <0.05 was considered statistically significant.

### Ethical considerations

The study was conducted in compliance with the ethical standards established by the institutional review board (IRB) of the University of Occupational and Environmental Health. Ethical approval was obtained before data collection, and the requirement for informed consent was waived due to the retrospective nature of the study and anonymization of patient data.

## Results

### Clinical characteristics and risk factors for DVT after cellulitis

The rationale for this analysis stemmed from prior evidence suggesting that cellulitis may increase the risk of thrombosis, potentially through the pro-inflammatory environment due to the local skin inflammation of cellulitis. To investigate the clinical characteristics associated with the development of DVT in the lower extremities following cellulitis, we hypothesized that inflammation induced by cellulitis could predispose patients to thrombosis, especially in the affected limbs. To explore this hypothesis, we evaluated key clinical parameters, including age, gender, BMI, CRP, D-dimer levels, the presence of malignancy, the use of corticosteroid, and type 2 diabetes. These factors were selected based on their well-established association with thrombosis in the existing literature.

**FIGURE 1:**
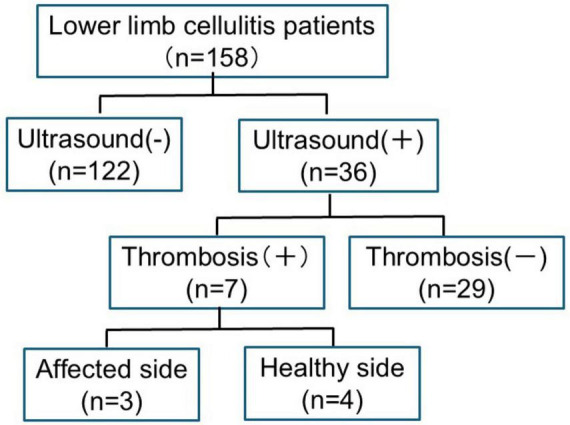
Flowchart of case selection for the study on thrombosis in cellulitis patients. This flowchart illustrates the selection process of patients included in the study. A total of 158 patients with cellulitis were initially screened. Among them, 36 patients underwent ultrasound examination due to elevated D-dimer levels (≥0.5 μg/ml). Of these, 7 cases were diagnosed with deep vein thrombosis (DVT), while 29 cases did not develop thrombosis.

Figure 1 outlines the patient selection process. A total of 158 cellulitis patients were initially screened, of which 36 were included in the final analysis based on the predefined inclusion and exclusion criteria. The screening involved assessing D-dimer levels, with patients presenting a D-dimer value ≥0.5 μg/ml undergoing Doppler ultrasonography to confirm the presence of DVT. Out of the 36 patients included, 7 (19.4%) were diagnosed with DVT, while 29 (80.6%) did not develop thrombosis.

Since inflammation is known to play a crucial role in thrombosis formation, CRP levels were evaluated to assess the systemic inflammatory status in the 36 patients (Table 1). The average CRP level was 10.5 mg/L in patients with DVT and 13.1 mg/L in those without DVT, with no significant difference observed (*P* = 0.542). Although CRP levels were not significantly different between the two groups, this finding must be viewed in light of the limited statistical power due to the small sample size. It remains possible that systemic inflammation contributes to thrombosis risk, but the current study may have lacked sufficient power to detect such an effect.

D-dimer levels, a widely recognized marker for detecting thrombosis, were also analyzed. All patients included in the study had elevated D-dimer levels (≥0.5 μg/ml) as part of the inclusion criteria. Within this selected cohort, the average D-dimer level was slightly higher in patients with DVT (6.27 μg/ml) compared to those without DVT (5.69 μg/ml), but this difference was not statistically significant (*P* = 0.832). While all patients had elevated D-dimer levels, and no significant difference was observed between those with and without DVT, this does not preclude a potential association. The limited sample size may have obscured a true relationship, and larger studies are needed to clarify the predictive value of higher D-dimer levels.

Obesity is another well-documented risk factor for thrombosis due to its contribution to venous stasis. To explore this, BMI was compared between the groups. The average BMI was 25.9 among patients with DVT and 30.8 among those without DVT, with no statistically significant difference (*P* = 0.227). Although no statistically significant difference in BMI was found between the two groups, this result should be interpreted with caution. Given the established link between obesity and thrombosis, further research with larger sample sizes is warranted to assess whether BMI plays a role in cellulitis-associated DVT.

Malignancy, often associated with a prothrombotic state, was also evaluated. Among patients with DVT, 28.6% (2 out of 7) had a history of malignancy, compared to 10.3% (3 out of 29) of those without DVT. Although the proportion of malignancy was higher in patients with DVT, this difference did not reach statistical significance (*P* = 0.224), suggesting a limited impact of malignancy on thrombosis risk in this study population.

Finally, we assessed the use of corticosteroids, which is often implicated in increasing thrombosis risk. Corticosteroid use was observed in 28.6% (2 out of 7) of patients with DVT and 27.6% (8 out of 29) of those without DVT, with no significant difference between the groups (*P* > 0.999). These findings indicate that corticosteroids had no measurable impact on thrombosis risk in cellulitis patients within this study.

Among the analyzed variables, the most notable finding was the significant association between age over 70 and an increased frequency of DVT. The mean age of the DVT-positive group was 78.7 years (range: 61–89), significantly higher than that of the DVT-negative group, which had a mean age of 66.3 years (range: 37–84) (*P* = 0.023). This unexpected result suggests that age rather than the degree of inflammation or other clinical parameters may be a key factor in thrombosis development in this population. The observed relationship between advanced age and thrombosis risk warrants further investigation, as it may be previously underappreciated.

### Multivariate analysis identifies age as an independent predictor of DVT

Following the unexpected finding that age may be a critical factor in thrombosis development, further analyses were conducted to evaluate the risk factors for DVT in cellulitis patients. A univariate analysis was first performed to explore potential associations between individual clinical variables and thrombosis risk (Table 2).

**TABLE 2:** The univariate and multivariate analysis.

	Univariate analysis		Multivariate analysis	
**Variables**	**Odds ratio (range)**	***P*-value**	**Odds ratio (range)**	***P*-value**
Age over 70	11.4 (1.2–108.3)	0.034	13.539 (1.109–165.334)	0.049
Sex	2.0 (0.3–12.2)	0.439	0.681 (0.030–15.648)	0.810
BMI	0.7 (0.1–3.8)	0.680	0.540 (0.046–6.316)	0.970
CRP	1.6 (0.2–15.6)	0.703	3.173 (0.143–70.618)	0.466
D-dimer	2.7 (0.3–25.8)	0.389	0.742 (0.019–29.475)	0.874
Diabetes mellitus	1.7 (0.3–9.0)	0.554	0.868 (0.087–8.627)	0.904
Malignancy	3.5 (0.5–26.4)	0.230	2.718 (0.212–34.899)	0.443
Corticosteroid use	1.1 (0.2–6.6)	0.958	0.945 (0.089–8.627)	0.963

The analysis revealed that patients aged 70 years or older had a significantly higher odds ratio (OR) of 11.4 (95% CI: 1.2–108.3, *P* = 0.034), indicating a notable association with DVT. However, other variables did not demonstrate statistically significant associations. For instance, sex (OR: 2.0, 95% CI: 0.3–12.2, *P* = 0.439) and BMI (OR: 0.7, 95% CI: 0.1–3.8, *P* = 0.680) showed no significant differences between patients with and without DVT. Similarly, CRP levels (OR: 1.6, 95% CI: 0.2–15.6, *P* = 0.703) and D-dimer levels (OR: 2.7, 95% CI: 0.3–25.8, *P* = 0.389) did not emerge as significant factors in this analysis. Other variables such as diabetes mellitus (OR: 1.7, 95% CI: 0.3–9.0, *P* = 0.554), cancer (OR: 3.5, 95% CI: 0.5–26.4, *P* = 0.230), and corticosteroid use (OR: 1.1, 95% CI: 0.2–6.6, *P* = 0.958) were also analyzed but did not show statistically significant associations. These results suggest that, within this cohort, none of these factors played a major role in thrombosis risk compared to age.

To confirm whether age was an independent risk factor, a multivariate analysis was performed, adjusting for the other clinical variables (Table 2). This analysis further supported the significance of age, with patients aged 70 years or older showing an adjusted OR of 13.5 (95% CI: 1.1–165.3, *P* = 0.049). Other variables, including sex (OR: 0.68, 95% CI: 0.03–15.65, *P* = 0.810), BMI (OR: 0.54, 95% CI: 0.05–6.32, *P* = 0.970), CRP (OR: 3.17, 95% CI: 0.14–70.62, *P* = 0.466), D-dimer (OR: 0.74, 95% CI: 0.02–29.48, *P* = 0.874), diabetes mellitus (OR: 0.87, 95% CI: 0.09–8.63, *P* = 0.904), cancer (OR: 2.72, 95% CI: 0.21–34.90, *P* = 0.443), and corticosteroid use (OR: 0.95, 95% CI: 0.09–8.63, *P* = 0.963), remained non-significant.

These findings confirm that age, specifically 70 years or older, is an independent predictor of thrombosis risk in cellulitis patients. While the exact mechanisms underlying this association remain unclear, the results emphasize the importance of increased vigilance in elderly patients during cellulitis treatment. Further research is required to elucidate the factors contributing to this observed relationship.

### Case-by-case analysis of thrombosis patients

**FIGURE 2:**
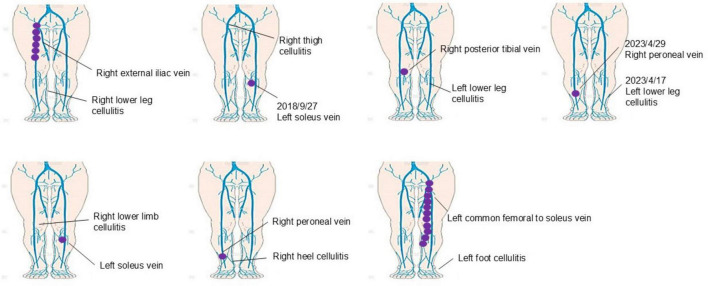
Case-by-case analysis of thrombosis in cellulitis patients. This figure presents a detailed case-by-case analysis of seven patients who developed thrombosis during cellulitis treatment. Thrombosis was observed in multiple venous sites, including the iliac vein (one case), peroneal vein (three cases), soleus vein (two cases), and one case involving thrombosis extending from the common femoral vein to the soleus vein. Notably, thrombosis occurred in the limb affected by cellulitis in three cases (42.9%), while four cases (57.1%) involved the contralateral limb. These findings highlight the diverse locations of thrombosis in cellulitis patients and emphasize the need for comprehensive vascular monitoring beyond the affected limb to ensure early detection and appropriate management.

A detailed analysis of seven patients who developed thrombosis during cellulitis treatment demonstrated the clinical variability and complexity involved in managing these cases as shown in Figure 2. We also examined the time interval between the diagnosis of cellulitis and the diagnosis of thrombosis in these seven patients. The duration ranged from 5 to 47 days, with a mean of 17.4 days and a median of 7 days. Thrombotic events were observed in multiple venous sites among the seven cases analyzed. Specifically, thrombosis occurred in the iliac vein (one case), peroneal vein (three cases), soleus vein (two cases), and one case where the thrombosis extended from the common femoral vein to the soleus vein. This analysis highlights the diverse locations of thrombosis in cellulitis patients, emphasizing the importance of thorough vascular monitoring to detect thrombotic events comprehensively. Of these thrombotic events, three cases (42.9%) occurred in the limb affected by cellulitis, while four cases (57.1%) involved the contralateral limb, suggesting that comprehensive vascular monitoring beyond the affected limb is required. This analysis demonstrates the diverse clinical presentations of thrombosis in cellulitis patients, underscoring the importance of thorough vascular monitoring and individualized treatment.

## Discussion

In this study, we investigated the clinical characteristics and risk factors associated with DVT in patients following cellulitis. Our findings provide important insights into the potential mechanisms of thrombosis formation in this context; however, they also raise new questions, particularly regarding the role of age in increasing the risk of thrombosis in cellulitis.

One of the most notable results from our study was the significant association between age (70 years or older) and the development of DVT. This finding was unexpected and highlights age as an independent risk factor for thrombosis in cellulitis patients, distinct from other commonly considered factors such as BMI, CRP levels, or malignancy. There are several possible explanations for why thrombosis was more frequently observed in older patients. First, aging is associated with increased vascular stiffness and decreased venous return, which could predispose elderly individuals to venous stasis, a known risk factor for thrombosis (15, 16). Additionally, older patients are more likely to have comorbid conditions, such as impaired mobility, which may further contribute to venous stasis during periods of immobility required for cellulitis treatment (17, 18). Another consideration is that the aging process is linked to a pro-inflammatory state, which might further promote thrombosis formation (19). However, our study did not find a significant association between inflammation (as measured by CRP) and thrombosis, suggesting that factors beyond systemic inflammation may be involved in older patients. Future studies are needed to explore the specific physiological changes that occur with aging and their role in increasing thrombosis risk in cellulitis patients.

Several limitations of this study should be acknowledged. First, the sample size may not have been large enough to detect subtle differences in certain clinical parameters, such as BMI, CRP levels, or the use of corticosteroid. Although we did not observe significant associations between these factors and thrombosis, it is possible that a larger cohort would reveal more nuanced relationships. Therefore, the absence of statistically significant differences in CRP, BMI, or D-dimer should not be interpreted as definitive evidence of no association. Rather, these findings likely reflect the limited statistical power of our sample and should be interpreted with caution. Additionally, this study was retrospective in nature, which may introduce selection bias and limit the generalizability of the findings.

Another limitation is that we used CRP as the primary marker for inflammation. While CRP is a well-established marker of systemic inflammation (20), it may not fully capture the complexity of the inflammatory response in cellulitis patients. In addition, our study found no significant difference in thrombosis formation between the affected and non-affected limbs. These findings suggest that neither localized inflammation nor systemic inflammation, as traditionally measured by CRP, may be the dominant driver of thrombosis in cellulitis patients. This raises questions about the role of alternative mechanisms beyond conventional inflammatory markers. For instance, in addition to the aging-related vascular changes, other inflammatory mediators not captured by CRP, such as IL-6, may play a more direct role in promoting thrombosis mediated by the positive action of thrombogenesis (21).

Despite the lack of significant associations with other clinical parameters, the findings of this study emphasize the importance of conducting further analyses. For example, although we did not find a significant association between BMI and thrombosis, obesity is a well-known risk factor for venous thromboembolism (22). It is possible that other variables, such as the duration of immobility or the use of certain medications, might interact with BMI to influence thrombosis risk. Future research is needed to explore these potential interactions. Although we did not find significant differences related to malignancy or the use of corticosteroid, these factors are known to predispose individuals to thrombosis in other settings (8, 23). The absence of significant findings in our study does not exclude the possibility that these factors contribute to thrombosis associated with cellulitis.

One of the key limitations of this study is the small sample size, with only 36 patients included and 7 cases of DVT identified. This limited number of outcome events reduces the statistical power to detect significant associations, particularly for less prevalent risk factors. As a result, there is a possibility of type II errors, and caution is warranted when interpreting non-significant findings. Despite these constraints, we proceeded with multivariate logistic regression analysis in an exploratory manner to adjust for potential confounding factors and to identify signals that could inform future, larger studies. The findings should therefore be considered hypothesis-generating, rather than definitive evidence of risk relationships.

A further consideration is the ultrasound screening protocol, which restricted Doppler imaging to patients with D-dimer levels ≥0.5 μg/ml. While this approach aligns with standard institutional practice, it may have resulted in underdetection of DVT cases with lower D-dimer levels, particularly in patients with isolated distal thrombosis or those with atypical presentations. As a result, the actual prevalence of DVT in our cohort may have been underestimated, and this potential selection bias should be considered when interpreting our findings.

Despite these limitations, our findings highlight the importance of considering age as a critical factor when evaluating DVT risk in cellulitis patients, and support the need for future prospective studies with larger cohorts to validate these observations.

Given that 57.1% of thrombosis cases occurred in the contralateral limb, comprehensive vascular monitoring beyond the affected limb should be considered, particularly in elderly patients with cellulitis. Clinicians should maintain a high index of suspicion for DVT in elderly cellulitis patients, even in the absence of significant inflammatory markers. Routine screening of both limbs may improve early detection and optimize management strategies.

## Data Availability

The raw data supporting the conclusions of this article will be made available by the authors, without undue reservation.
